# Prevention and Treatment of Phlebitis Secondary to the Insertion of a Peripheral Venous Catheter: A Scoping Review from a Nursing Perspective

**DOI:** 10.3390/healthcare9050611

**Published:** 2021-05-19

**Authors:** Aitana Guanche-Sicilia, María Begoña Sánchez-Gómez, María Elisa Castro-Peraza, José Ángel Rodríguez-Gómez, Juan Gómez-Salgado, Gonzalo Duarte-Clíments

**Affiliations:** 1 University Hospital Nuestra Señora de Candelaria, Canary Islands Health Service, 38010 Tenerife, Spain; aitanaguanche@gmail.com; 2 University School of Nursing Nuestra Señora de Candelaria, University of La Laguna, 38001 Tenerife, Spain; extmsanchez@ull.edu.es (M.B.S.-G.); mcastrop@ull.edu.es (M.E.C.-P.); extgduartcl@ull.edu.es (G.D.-C.); 3 Chair of Nursing, Faculty of Health Sciences, University of La Laguna, 38001 Tenerife, Spain; jarogo@ull.edu.es; 4 Department of Sociology, Social Work and Public Health. Faculty of Labour Sciences, University of Huelva, 21007 Huelva, Spain; 5 Safety and Health Postgraduate Programme, Universidad Espíritu Santo, 092301 Guayaquil, Ecuador

**Keywords:** phlebitis, catheters, nursing care, patient-centered care, patient safety, evidence-based practice

## Abstract

The objective of this work was to identify available evidence on nursing interventions for the prevention and treatment of phlebitis secondary to the insertion of a peripheral venous catheter. For this, a scoping systematic review was carried out following the guidelines in the PRISMA declaration of documents published between January 2015 and December 2020. The search took place between December 2020 and January 2021. Scielo, Pubmed, Medline, Scopus, WOS, CINHAL, LILACS, and Dialnet databases were consulted, and CASPe, AGREE, and HICPAC tools were used for the critical reading. A total of 52 studies were included to analyze nursing interventions for treatment and prevention. Nursing interventions to prevent phlebitis and ensure a proper catheter use included those related to the maintenance of intravenous therapy, asepsis, and choosing the dressing. With regard to the nursing interventions to treat phlebitis, these were focused on vigilance and caring and also on the use of medical treatment protocols. For the prevention of phlebitis, the highest rated evidence regarding asepsis include the topical use of >0.5% chlorhexidine preparation with 70% alcohol or 2% aqueous chlorhexidine, a proper hygienic hand washing, and the use clean gloves to handle connections and devices. Actions that promote the efficacy and safety of intravenous therapy include maintenance of venous access, infusion volume control, verification of signs of phlebitis during saline solution and medication administration, and constant monitoring. It is recommended to remove any catheter that is not essential. Once discharged from hospital, it will be necessary to warn the patient about signs of phlebitis after PVC removal.

## 1. Introduction

Vascular access cannulation through the use of peripheral venous catheters (PVCs) is a common practice and is considered the most common invasive procedure performed on hospitalized patients [1]. This technique allows quick access to the vascular system, being less invasive and less complex than other techniques [1,2,3]. The type of catheter is chosen based on the estimated duration and type of treatment to be infused, and among the uses of PVCs are fluid therapy, parenteral nutrition, blood products, and diagnostic tests [4].

The most common side effect of PVC is phlebitis [5]. This consists of acute inflammation of the wall of the blood vessels, with irritation of the venous endothelium in the section or segment cannulated by the catheter [6]. Identification of phlebitis requires assessment of possible signs and symptoms present in the insertion area, such as erythema, tumefaction in the vein, pain, heat, and fever [7]. In this sense, the use of rating scales such as the Visual Infusion Phlebitis (VIP) scale, the Phlebitis scale, and the Maddox scale [8,9,10] may be useful.

In Spain, up to 30% of the bacteriaemia associated with hospital care is related to the use of intravascular devices, and these produce increased morbidity and hospital expenses, which are estimated at around 18,000 euros per episode [4].

PVC management is an under-recognized issue regarding patient risk within the complexity of care [11]. It was verified through the National Study of Adverse Events (ENEAS, for its acronym in Spanish) that if phlebitis is included as an adverse healthcare event, it would rank first followed by medication errors, infections arising from healthcare-related practices, and surgical techniques [7]. On the other hand, the SENECA Project: Quality of care standards for patient safety in National Health System hospitals, enabled from 1344 revised medical records, from 32 different hospitals, identified approximately 377 patients who developed phlebitis and/or extravasation (25.1%) [7]. Phlebitis prolongs hospitalization and treatment, increases economic expenses, decreases patient satisfaction, and can lead to other complications such as sepsis, pain, discomfort, stress, possibility of clotting, thrombophlebitis, and embolism [5,12,13].

The Infusion Nurses Society (INS) [9] indicates that the accepted phlebitis rate should be 5% or less [14,15]. Currently, a phlebitis incidence of 0.5% to 59.1% [5] is estimated, with a prevalence of between 20 and 80% of patients following intravenous treatment [16].

Nurses’ knowledge on the proper management of PVC and early recognition of risk factors can reduce these complications [14,17]. In Spain, it is a nursing competence, collected in Order CIN/2134/2008, of 3 July [18]. In addition, the North American Nursing Diagnosis Association (NANDA-I) collects a risk diagnosis to prevent risk of (00213) phlebitis, and the Nursing Intervention Classification (NIC) establishes interventions for the prevention and treatment of phlebitis such as (4200) Intravenous therapy (iv); (2314) Medication administration: intravenous (iv); and (4235) Phlebotomy: Cannulated Vessel [19,20].

For this reason, the objective of this review was to identify available evidence on nursing interventions for the prevention and treatment of phlebitis secondary to the insertion of a peripheral venous catheter.

## 2. Materials and Methods

### 2.1. Design 

A systematic scoping review [21] of the literature was carried out, following the standards set out in the PRISMA extension for Scoping Reviews (PRISMA-ScR) [22]. Based on the PICO format (Table 1), the following research question was developed: *What are the evidence-based nursing interventions for the prevention and treatment of phlebitis in patients with PVC?*

### 2.2. Search Strategy 

The search was performed between December 2020 and January 2021 (Table 2). The databases consulted were Scielo, Pubmed, Medline, Scopus, WOS, CINHAL, LILACS, and Dialnet using MeSH and DeCS descriptors (Table 2).

### 2.3. Selection Criteria

The search was limited to full-text documents, in Spanish, English, and/or Portuguese, published in the last 5 years. These included empirical studies and clinical guidelines that focused on phlebitis, its classification, diagnosis, prevention, and treatment from the nursing competence perspective. Those that, during the critical reading, did not meet the expected criteria in the first three questions of the CASPe [23] tool nor of Berra et al.’s instrument [24] for descriptive articles were excluded. Articles not available in full text and published in languages other than Spanish, English, or Portuguese were also excluded.

Following the recommendations for improvement for Scoping Reviews by Levac, Colquon, and O’Brien [25], it was decided not to discriminate by type of study in the selection. In the classification and presentation of results, we differentiate according to the design of the selected studies.

### 2.4. Data Collection and Extraction

The search was conducted independently and pooled by the researchers as detailed in the Authors Contributions. The authors performed an evidence synthesis by peer evaluation, analyzing each intervention and evidence individually, following the criteria provided by the Joanna Briggs Institute (JBI). The discrepancies were jointly discussed to reach consensus on the degree of evidence (DE) and the degree of recommendation (DR).

### 2.5. Evaluation of Methodological Quality

Once the titles and summaries of the articles were critically read, data were classified according to the objectives of the study. The included full-text articles were assessed with the CASPe [23] and Berra et al.’s [24] tools. For clinical practice guidelines, AGREE [26] was used for critical reading and the HICPAC system for evidence synthesis [27]. The JBI [28] criteria were used to classify the degree of evidence (DE) and the degree of recommendation (DR) of nursing interventions.

## 3. Results

**Figure 1:**
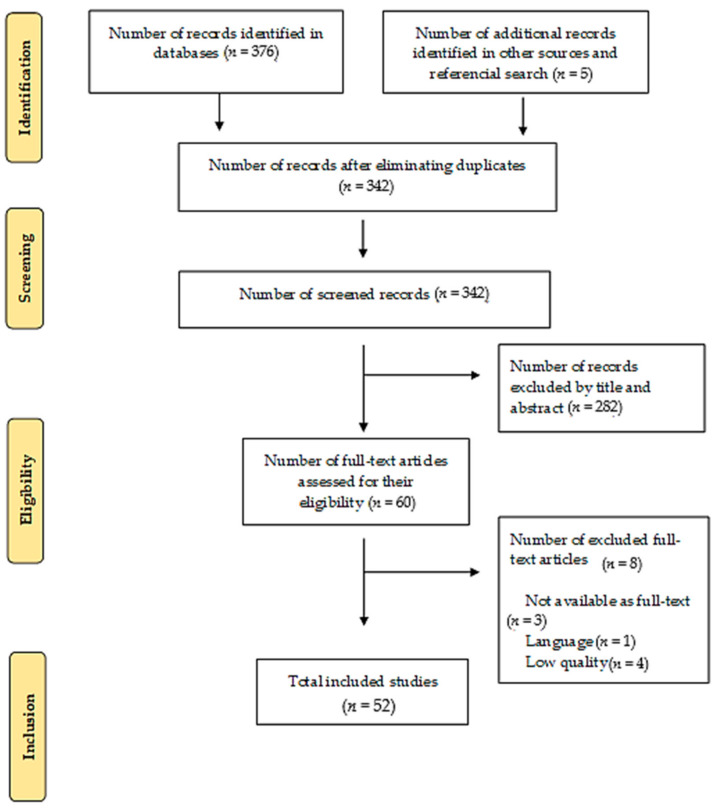
Search results (PRISMA flowchart).

The number of records identified in the databases was 376, to which five more records were added by referential search to introduce the clinical practice guidelines, for a total of 381 references. Of the 381 identified references, 52 studies were finally selected according to eligibility criteria (Figure 1) [22].

In terms of typology, 9 randomized clinical trials, 17 cohort studies, 8 systematic reviews, 3 case and control studies, 1 qualitative study, 10 descriptive studies, and 4 clinical practice guidelines, out of the total of 52 selected studies were included. Table 3 lists the characteristics of the studies included in the scoping review classified according to the type of article.

The articles are classified as nursing interventions for the prevention of phlebitis or for the treatment of phlebitis, both for nurses who apply care independently and for nurses that follow guidelines and protocols.

### 3.1. Nursing Interventions for the Prevention of Phlebitis

Management and maintenance of intravenous therapy

- Protocol monitoring and continuous evaluation [28,29].
- Records need to include date of puncture, dressings used, professional performing the procedure, date of change of PVC, puncture locations, number of puncture attempts, intravenous medication in use, change of dressing, and next check-up [4,44].
- For intermittent flushing and locking of PVC, it is recommended to use saline solution (SS) before and after administration of medication by performing the positive pressure technique [4,29]. According to the Flebitis Zero project, flushing heparin is not recommended because it can cause thrombocytopenia [7]. A randomized clinical trial reflected that continuous infusion of heparin into PVCs improved the duration of permeability, reducing infusion failure and phlebitis. However, no statistically significant differences were found when heparin was used intermittently. If PVC is used to obtain a blood sample, the use of diluted heparin is indicated [43]. Double-pump syringes enable both medication and cleaning solution administration to reduce PVC manipulation and complications [40].
- To prevent and treat phlebitis, Aloe vera, Matricaria chamomilla, and Xianchen (composed of some components such as Fritillaria and Bletilla striata) can be used. The application of Moist Exposed Burn Ointment (MEBO) for topical treatment of burn injuries, containing sesame oil, β-sitosterol, Berberine, and other medicinal plants (Coptis chinensis, Scutellaria baicalensis, Phellodendron chinese, and Papaver somniferum) [36] is also effective.
- Remove peripheral venous catheters if the patient develops signs of phlebitis (warmth, tenderness, erythema, or palpable venous cord), infection, or a malfunctioning catheter [7,45].
- Not performing a systematic catheter change every 72 to 96 h. It must be changed when clinically justified. There are no significant differences in the percentage of complications (phlebitis, occlusion...) between PVC that have been inserted less than 96 h and those that have been inserted for more than 96 h [4,46,47].
- Avoiding insertion into joint areas, wrist, and antecubital fossa, because there is a higher incidence of mechanical phlebitis related to catheter movement [4].
- Replacing administration systems, extension cords, and accessories with a frequency of more than 96 h and less than 7 days, when dirty or damaged connections are observed, and whenever there is an accidental disconnection of the circuit [4].
- Withdrawing systems of blood administration and of blood products at the end of transfusion [4].
- In adults, an upper-extremity site for catheter insertion must be used. Any catheter inserted in a lower extremity must be replaced to an upper extremity site as soon as possible [4].
- Using a 0.22-micron line filter to remove air and bacteria and small drug particles that were not properly diluted. A filter is also required when the infusion of amiodarone exceeds 24 h [48].
- Guiding patients and family members on signs and symptoms of phlebitis, during infusion and post-infusion after extraction of PVC [2,31]. Educating patients on the fact that ensuring proper care of PVC helps reduce risk of infection [17].

### 3.2. Asepsis

- Using alcoholic chlorhexidine solution at > 0.5% or aqueous chlorhexidine at 2%. In cases of hypersensitivity, iodine solutions or alcohol at 70% may be used [4,17].
- Applying antiseptics to clean skin and complying with drying times (2% alcoholic chlorhexidine: 30 s; non-alcoholic chlorhexidine and povidone–iodine: 2 min) [4].
- Hygienic hand washing and usage of clean gloves for both punctures and equipment, hubs, stopcocks, and bio-connectors handling. It is not necessary to wear sterile gloves if the previously disinfected area is not touched again during the technique [4]. Using disposable tourniquets can help reduce PVC contamination rates [49].
- Minimizing handling of connectors for infusion equipment [4].
- Protecting dressing and connectors in activities that may pose a risk of contamination [4].
- High incidence of phlebitis and infection of inserted PVCs has been found in emergency areas. In these cases, it is recommended to replace the catheter within the first 48 h if aseptic technique could not be ensured [7,41].

### 3.3. Nursing Assessment

- Involving the patient in the choice of PVC and puncture site [2].
- Analyzing patient characteristics, prescribed medications (irritant and/or vesicant, pH, and osmolarity), expected duration of the treatment, and other risk factors for the onset of phlebitis, before opting for a PVC [4,30].
- Assessing the status of venous resources. Whenever possible, choose straight, palpable, and well-filled vessels [4].
- Keeping the PVC insertion site visible [40,45]. Developing an observation table to document the development of signs of phlebitis for early detection and decreased discomfort and pain [50].
- Previously identifying comorbidities such as diabetes mellitus due to the changes in the circulatory system caused by this disease [31].
- Asking the patient if there is pain/discomfort, heat, or burning at the insertion site [32].

### 3.4. Catheter

- Selecting the length and caliber of the catheters based on objective, expected time of use, known infectious or non-infectious complications, experience of those who insert and manage the catheter [7].
- Selecting a catheter of the least length and caliber possible, not exceeding the caliber of the chosen vessel, to allow blood to pass into the vessel and favor the hemodilution of the preparations to be infused [4,45].
- Using the minimum number of three-way stopcocks. Idle ports should always be capped [4].
- Using only one of the ports of the three-way stopcock to place a bio-connector, where medication solutions and bolus will be administered. The results of a prospective experimental study indicate that using SwabCap significantly reduced connector contamination from 43.7% to 0% (*p* = 0.006) [51]. If this cap is not available, the bio-connector is disinfected with alcoholic chlorhexidine at >0.5% or 70% alcohol for 30 s [4,7].
- Teflon, silicone, or polyurethane elastomer catheters are safer than those of polyethylene, polyvinyl hydrochloride, or steel needles, which might cause tissue necrosis if extravasation occurs [43].

### 3.5. Dressing

- It is not advisable to bandage the site of the intravenous line. Sterile, transparent [45], semipermeable adhesive dressing will be used to improve visibility of the insertion site [7,32,52].
- The dressing should be placed aseptically, with clean or sterile gloves, without excessively touching the adhesive layer and without placing tie-shaped adhesive tapes under the dressing. Wear sterile gloves for central and arterial devices [4].
- Changing dressings at least every 7 days, except in pediatric patients, where the risk of moving the catheter is greater than the advantages derived from changing the dressing [7]. Routine dressing change is not recommended, as it increases the risk of colonization at the puncture site [49].
- Replacing catheter site dressing if the dressing becomes damp, loosened, or visibly soiled [7].
- If the site is bleeding or oozing, use gauze dressing until this is resolved [4].
- Ensuring correct securement or dressing to prevent dislodgement [4].
- Softly removing dressing, without moistening the puncture site [4].
- For catheter securement, products such as CliniFix simultaneously reduce the risk of infection and trauma from cannula movement. Made with hydrocolloid adhesive, not harmful to the skin, that can remain in place for up to 7 days without affecting the integrity of the skin. In the presence of wound oozing, hydrocolloids absorb fluid and form gel to help reduce the spread of infection [42].
- Using skin glue (cyanoacrylate) at the insertion site to improve catheter securement and reduce rates of phlebitis and occlusion. Apply a drop at the insertion site and a drop under the center of the catheter, allow to dry for 30 s, and place a dressing [33].
- Using the “I.V. House UltraDressing” in pediatric patients to increase catheter dwell time, and to protect and stabilize PIVCs [34].

### 3.6. Nursing Interventions for the Treatment of Phlebitis

Nurse as a care prescriber

- Apply alternating hot and cold compresses to decrease erythema, edema, and pain. The hot compress stimulates vasodilation by inducing optimal blood circulation and promoting a faster wound-healing process. The cold compress stimulates vasoconstriction and reduces edema [35].
- Apply compresses with 0.9% NaCl to stimulate anti-inflammatory response and relieve pain, redness, swelling, and edema [35].
- Apply 10 drops (3 mL) of sesame oil (SO) twice daily for two weeks. Massage for 5 min within 10 cm of the place of phlebitis. Use a finger with a sterile glove to apply a rotary technique. SO contains unsaturated fatty acids (linoleic acid, oleic acid) that relieve pain by reducing prostaglandins and leukotriene. In addition, the SO has lignans responsible for analgesic and anti-inflammatory effects [36].
- Administer chamomile extract (2.5%), as it has anti-inflammatory and anti-edema properties. It is the most effective in the treatment of grade II phlebitis. The use of topical chamomile showed that the incidence of phlebitis in patients treated with amiodarone decreases significantly. Aqueous chamomile extract inhibits the production of prostaglandins by the suppression of cyclooxygenase-2 (COX-2) and direct gene expression of inhibition of COX-2 enzyme activity [37].
- Apply a compress with Burow solution at a temperature between 2 and 8 degrees Celsius, and leave on for 20 min every 8 h. This formulation has as its main component aluminium acetate, which is known for its astringent properties and ability to produce the precipitation of proteins at the topical level. Based on observed results, the Burow solution could be defined as an effective therapeutic alternative in the treatment of post-infusion phlebitis [38].
- Apply marigold ointment every 8 h. Calendula/marigold flavonoids prevent histamine release and prostaglandin production. In addition, they inhibit blood plasma secretion in tissues, reduce the migration of white blood cells to the swelled area, and prevent the growth of bacteria and fungi by reducing capillary permeability. In one study, the application of marigold ointment decreased the severity of phlebitis in a shorter period compared to using a wet and hot compress. It is proven to have anti-inflammatory and antibacterial effects. Although calendula has an anti-inflammatory effect similar to corticoids, it has none of its complications and is safe to use [39].

### 3.7. Nurses Following Protocols and Guidelines

- Topical treatments with Aloe vera or “Chamomilla Recutita” using wet compresses at 38 degrees Celsius on the affected area. Apply topical diclofenac and "Essaven" heparin gel. Other topical products such as notoginseny and 5 mg nitroglycerin patch accelerate the improvement of phlebitis symptoms, as compared to heparinoid substances [6].
- Dilution of chemotherapeutic agents, immediate catheter removal, intermittent heparin washing, prophylactic antibiotics, transparent dressings, topical application of anti-inflammatory or corticosteroid agents, and application of a hot and/or wet compress are preventive and therapeutic approaches [36].
- There is no definitive treatment to prevent phlebitis; drugs such as heparin, corticosteroids, and piroxicam have been proposed as therapeutic agents [39].

In addition, Table 4 identifies the interventions found in the literature, their evidence synthesis, and their correspondence with NIC nursing interventions.

## 4. Discussion

The objective of this review was to identify available evidence on nursing interventions for the prevention and treatment of phlebitis secondary to the insertion of a peripheral venous catheter.

For the prevention of phlebitis, the greatest evidence found regarding asepsis is to use >0.5% chlorhexidine preparation with alcohol or 2% aqueous chlorhexidine, perform hygienic hand washing, and use clean gloves to handle connections and stopcocks (category AI). Regarding the maintenance of PVC, the interventions with the greatest evidence (category IA) are replacing extensions and administration sets between 4 and a maximum of 7 days if they are not loosened or soiled, and using the fewest three-way stopcocks, one of the ports with a bio-connector, and having the others capped [4]. Actions that promote the efficacy and safety of intravenous therapy include maintenance of access, infusion control, verification of signs of phlebitis during saline solution replacement and medication administration, and constant monitoring [53,54]. It is recommended to remove any catheter that is not essential (Category IA) [7]. Once discharged from hospital, it will be necessary to warn the patient about signs of phlebitis after PVC removal [2] (DE 1a).

Regarding the dressing, the greatest evidence obtained is the use of “IV House UltraDressing” in pediatric patients to increase dwell time and stabilize the catheter [34] (DE IA). The dressing must be sterile, transparent, and semi-permeable for continuous visual inspection of the catheter site [7] (Category IA). A novel technique is the use of medical grade cyanoacrylate for catheter securement to reduce rates of phlebitis [33] (DE 1c).

Vein quality control through palpation and visual inspection can prevent phlebitis as well as the nurse’s duration of hand hygiene and the clinical experience [55]. Regarding the choice of veins, a controversy has been found, as some studies recommend the forearm as it has a larger diameter and this reduces rates of phlebitis in the region, but there are many others that claim that this region should be avoided, in addition to the joints and wrist areas (category IA), for having a higher incidence of mechanical phlebitis related to catheter movement [4,56]. A review of the literature concluded that veins from the antecubital region are associated with lower rates of phlebitis, as compared to veins in the hands. Therefore, although the back of the hand is considered an easily accessible venous place, it is not indicated for prolonged venous therapy, and nurses are believed to need to be trained in carrying out alternatives such as jugular vein venipuncture that has very low rates of prevalence of phlebitis [44].

Although some studies, such as the one conducted by Circolini et al. (cited in Wei et al. [57]), in which Cox’s analysis was used, showed that as the PVC’s dwell time increased by 24 h, the risk of phlebitis also did so with an odds ratio of 1.05. Webster et al.’s [46] team concluded in their clinical trial review that the catheter should be removed only when clinically indicated, evaluating at every new shift to identify signs of phlebitis [46,57,58] early.

There are higher rates of phlebitis when larger caliber catheters are used, as they increase attraction to the vessel wall, so the smallest possible length and caliber catheters (Category IB) are recommended [4,56].

The nurse’s clinical judgment regarding the selection of a PVC should also involve assessing comfort, anxiety, and restrictions in the patient’s day-to-day activities. The number of venipuncture attempts is one of the quality indicators and shows patient satisfaction for its sensory impact [59].

For the control and management of intravenous therapy, nurses should carry out a protocol that includes recording all aspects related to the catheter and its maintenance (DE 4a and DR A) [28].

According to the studies consulted, the data show a higher proportion of complications in peripheral venous catheters among women (*p* = 0.0300), patients 85 years of age or older as compared to those under 65 (*p* = 0.0500), and when puncturing the forearm region as compared to other regions (*p* ≤ 0.0001) [60].

Regarding saline flush, the effectiveness and safety of a solution of 0.9% of sodium chloride was compared with heparin saline solution, and it was concluded that both agents are equally effective and safe [14]. However, flushing PVC with normal saline prevents the accumulation of bacteria, proteins, and platelets suspended in plasma; therefore, saline flush prevents and reduces phlebitis [13] (DE 1c).

It is necessary to check that the prescription of intravenous medication can be administered peripherally, as many of these drugs are irritating. Regular evaluation is key to the prevention and early detection of intravenous complications (DE 5a) [30,61].

In case the patient has phlebitis, nursing interventions with more evidence in their application are Moist Exposed Burn Ointment (MEBO) for topical treatment of burn injuries, Aloe vera, chamomilla recutita in wet compresses, topical diclofenac, “Essaven” heparin gel, notoginseny, and 5 mg nitroglycerin patch and anti-inflammatory or corticosteroids in a hot or wet compress (DE 1a) [6,36]. On the other hand, the application of marigold ointment, piroxicam, and sesame oil has an evidence level of 1C [36,39].

Some limitations of this bibliographic review are the little available evidence on the use of Burow’s solution as a treatment for phlebitis, despite its usual use in hospitals. This review may contain limitations inherent to the search and selection process. Therefore, we believe that further research and systematic review of the findings is needed.

## 5. Conclusions

In conclusion, this review includes evidence-based interventions for the prevention and treatment of phlebitis associated to the venous catheter. The need for nursing training on the latest available evidence regarding the use and management of venous catheters is highlighted. It is important that hospitals implement projects such as “Flebitis Zero” so that nurses can rely and base their knowledge on them, thus providing quality care to patients.

## Figures and Tables

**Table 1:** PICO.

Patient	Patients with Peripheral Venous Catheter
Intervention	Evidence-based nursing interventions for the prevention and treatment of phlebitis
Comparison	Non-evidence-based routine interventions
Outcomes	Use of sterile and transparent dressing, catheter size, use of hot and cold compresses, topical treatment, management and maintenance of intravenous therapy

**Table 2:** Search strategy.

Database	Date (dd/mm/yyyy)	Search Strategy	Found Articles	Pre-Selected Articles
Scielo	7/12/2020	Phlebitis AND catheter	19	10
Scielo	02/01/2021	Phlebitis AND treatment	5	1
Scopus	7/12/2020	Phlebitis and nurs*	20	6
Scopus	02/01/2021	Phlebitis AND treatment	6	1
Pubmed	2/12/2020	Phlebitis and nurs*	20	6
Pubmed	12/12/2020	Phlebitis AND intervention AND nurs*	2	1
Pubmed	21/12/2020	Phlebitis AND nurs* intervention	17	2
Pubmed	21/12/2020	Phlebitis AND assessment measure	1	1
Pubmed	13/01/2021	Phlebitis AND prevention AND nurs*	5	1
Pubmed	25/11/2020	Competence AND nurs* AND phlebitis	13	5
Dialnet	13/12/2020	Phlebitis AND nursing	13	3
Medline	13/12/2020	Phlebitis and nursing interventions	2	2
Medline	19/12/2020	Phlebitis and nurs*	64	8
Medline	19/12/2020	Phlebitis AND treatment OR intervention OR therapy AND nurs*	41	2
WOS	21/01/2021	Phlebitis AND nurs* AND prevention	15	5
WOS	21/01/2021	Phlebitis AND treatment AND nurs*	15	2
LILACS	21/12/2020	Phlebitis AND nursing	65	8
LILACS	21/12/2020	Phlebitis and intervention	3	1
CINHAL	02/01/2021	Phlebitis AND treatment OR intervention OR therapy AND nurs*	27	5
CINAHL	02/01/2021	Phlebitis AND intervention	23	1
Total			**376**	**71**

**Table 3:** Characteristics of the studies included in the scoping review.

a. RESULTS FROM CLINICAL STUDIES					
Year and Author	Objective	Type of Study	Results	Critical Reading	JBI Synthesis of the Evidence
Reichembach-Danski et al., 2016 [29]	Evaluate the incidence of complications related to peripheral intravenous catheter use in neonates and identify associated risk factors.	Prospective cohort study	Protocols with measures such as continuous observation of the insertion site, keeping the catheter insertion site visible, and saline solution infusion prior to the administration of other substances may be used to reduce complications.	9/11 ****	DE*: 3c DR: A
Braga et al., 2018 [30]	Determine the incidence rate and risk factors for phlebitis and infiltration in patients with peripheral venous catheter.	Cohort study	To improve quality of care and prevent phlebitis and infiltrations, the nurse should analyze patient characteristics, prescribed intravenous medications (irritant and/or vesicant, pH, and osmolarity), expected duration of intravenous treatment, and risk factors for complications that can occur before selecting a venous catheter. Evaluate risks and benefits of each type of catheter and consider patient preferences. This test may indicate the use of other venous catheters, such as peripherally inserted central catheter.	9/11 ****	DE*: 3c DR: A
Rosário-Pereira et al., 2019 [31]	Describe cases of phlebitis reported in a university hospital and contribute to possible improvement actions in quality of care and safety.	Retrospective, observational study	Knowing the characteristics of phlebitis favors understanding and minimization of this adverse event so as to establish preventive behaviors and decrease risk and incidence.	Intermediate evidence *****	DE*: 3e DR: A
Lim et al., 2018 [32]	Project to improve visibility of intravenous area through the use of transparent adhesive dressings to achieve frequent and adequate intravenous inspection of the intravenous area and reduce incidence of complications.	Quasi-experimental study	Improved visibility of the intravenous puncture site and nurses’ compliance in frequent testing and monitoring; phlebitis can be detected early, reducing severity of complications.	8/11 ****	DE*: 2d DR: B
Bugden et al., 2016 [33]	Assess whether the use of a skin adhesive glue to secure a peripheral intravenous line improves failure rates compared with standard securing measures	Randomized clinical trial	Adding a drop of cyanoacrylate adhesive helps stabilize the peripheral intravenous catheter.	9/11 ****	DE*: 1c DR: A
Büyükyılmaz et al., 2019 [34]	Evaluate efficacy of I.V UltraDressing to protect peripheral intravenous catheters in pediatric patients.	Randomized clinical trial	I.V House UltraDressing device is useful to increase catheter dwell time and protect and stabilize PVCs in pediatric patients.	9/11 ****	DE*: 1c DR: A
Annisa et al., 2017 [35]	Assess the effectiveness of applying hot compress to reduce the degree of phlebitis.	Quasi-experimental study	A hot water compress is useful in the treatment of phlebitis and could decrease degree of phlebitis in an effective and cost-saving way.	8/11 ****	DE*: 2d DR: A
Bigdeli-Shamloo et al., 2019 [36]	Assess the effects of topical sesame oil on reduced severity of "chemotherapy-induced phlebitis" pain.	Randomized clinical trial	Complementary methods for the treatment of phlebitis symptoms are proposed. Application of sesame oil is effective in reducing severity of chemotherapy-induced phlebitis pain.	9/11 ****	DE*: 1c DR: A
Sharifi-Ardani et al., 2017 [37]	Assess the effect of topical chamomile on phlebitis caused by the administration of amiodarone infusion in PVC.	Randomized clinical trial	Topical chamomile may be effective in decreasing incidence of phlebitis for its anti-inflammatory capacity.	9/11 ****	DE*: 1c DR: A
García-Quintanilla et al., 2018 [38]	Describe the use and assess the efficacy and safety of Burow solution for topical treatment of phlebitis.	Prospective observational study	During the inclusion period for Burow solution in 35 patients with phlebitis, 12 have been excluded for lack of follow-up. 83% (n = 19) have responded to the therapy by reducing the degree of phlebitis by two points after three days of treatment, and 17% (n = 4) were unresponsive, requiring topical applications of Feparil®.	7/11 ****	DE*: 3e DR: A
Jourabloo et al., 2017 [39]	Compare the effect of wet compresses and marigold ointment on the severity of phlebitis caused by dextrose infusion.	Randomized clinical trial	Applying marigold ointment decreased the severity of phlebitis in a shorter period, compared to using a wet, hot compress. This ointment is recommended to reduce phlebitis severity.	9/11 ****	DE*: 1c DR: A
Parreira et al., 2020 [40]	Establish clinical efficacy and safety parameters for double-pump syringes for intravenous medication administration and PVC washing.	Randomized clinical trial	Developing a double-pump syringe makes it easier for nurses to adopt good clinical practices in procedures and administer intravenous medicine to reduce catheter handling.	8/11 ****	DE: 1c * DR: A
Pérez-Granda et al., 2020 [41]	Compare rates of phlebitis and catheter tip colonization between PVC blocked with saline solution and blocked with heparin in patients admitted to internal medicine.	Randomized clinical trial	No statistically significant differences were found in frequency of phlebitis and PVC tip colonisation regarding blockage with saline solution or with heparin. PVC can be maintained with saline solution, and it is safer and cheaper than heparin.	8/11 ****	DE: 1c * DR: A
**b. RESULTS FROM REVIEWS**					
**Year and Author**	**Research Question**	**Methodology**	**Results**	**Critical Reading**	**JBI Synthesis of the Evidence**
Higgingson, 2015 [42]	Assess complications associated with improperly secured intravenous cannulae, along with examination of fastening devices.	Literature review	Intravenous catheters not secured properly produce higher rates of mechanical phlebitis and infection. Clinical staff need to be educated on PVC stabilization as an important measure to reduce phlebitis. Safety devices are available for their use whenever appropriate.	8/11 ****	DE*: 4a DR: A
Chau and Pellowe, 2008 [43]	Provide summarized evidence on intravascular device management to reduce risk of infection.	Best practice information sheet	Continuous training and evaluation are needed on handling, aseptic technique and hand washing, choosing a catheter based on estimated function, duration, and complications.	Recommended ***	DR: A *
Chau and Pellowe, 2008 [43]	Provide summarized evidence on intravascular device management to reduce risk of infection.	Best practice information sheet	Flush stopcocks with saline solution.	Recommended ***	DR: B *
Martín-Gil et al., 2017 [6]	Effectiveness of topical treatments in hospitalized patients with phlebitis secondary to peripheral venous catheterisation to improve or resolve signs and symptoms.	Systematic review of clinical trials and reviews	Aloe vera, notoginseny, diclofenac, and heparin gel 1000 IU showed a level of evidence and a moderate degree of recommendation. Heparin gel is the only compound indicated by the Spanish Agency of Medicines and Medical Products to treat post-infusion phlebitis; notoginseny is not marketed in the Western world; and diclofenac is an anti-inflammatory used in various pathologies.	10/11 ****	DE*: 1b DR: A
**c. RESULTS FROM QUALITATIVE STUDIES**					
**Year and Author**	**Research Question**	**Methodology**	**Results**	**Critical Reading**	**JBI Synthesis of the Evidence**
Salgueiro-Oliveira et al., 2019 [2]	What are the PVC-related nursing practices for identifying deviations from available evidence on phlebitis prevention?	Qualitative study, participants monitoring and interviews	Nursing practices that differ from scientific evidence; influence from institutional dimensions; characteristics of the sick and misinformation about patient safety risk actions. Developing protocols and implementing continuing education are critical to acquiring skills, correcting and providing safe and quality assistance.	9/11 ****	DE*: 4b DR: A
**d. RESULTS FROM DESCRIPTIVE STUDIES**					
**Year and Author**	**Research Question**	**Methodology**	**Results**	**Critical Reading**	**JBI Synthesis of the Evidence**
Da Silva-Oliveira et al, 2016 [44]	What are the characteristics of phlebitis reported in a hospital of the Sentinel Hospital Surveillance Network?	Descriptive quantitative study	For the prevention of phlebitis, an educational intervention through team training has the potential to reduce 50% of cases in peripheral intravenous therapy. Phlebitis rates/incidence are used as an indicator of nursing quality of care. As best prevention practices, it is suggested to use smaller calibre cannulae, transparent dressings, and professional knowledge of signs and symptoms that warn of possible phlebitis. On the venous access site, a statistical association was found between the back of the hand with more grade I phlebitis, followed by antecubital pit with higher number of grade II and grade III phlebitis. It is advisable to prioritize peripheral catheterisation in upper limb blood vessels, as they are safer. Avoid joint areas with greater mobility, more prone to traumatic mechanical phlebitis formation, and control osmolarity and pH of medication to reduce chemical phlebitis.	HIGH evidence *****	DE*: 4a DR: A
**e. RESULTS FROM CLINICAL PRACTICE GUIDELINES**					
**Year and Author**	**Research Question**	**Methodology**	**Results**	**HICPAC Category**	**AGREE**
Torres-Muñoz et al., 2018 [4]	Guide to recommendations on nursing care of vascular accesses.	Clinical Practice Guideline	PVC insertion technique: Handwash with antiseptic soap and water if visibly dirty (40–60 s) or alcoholic-based friction (20–30 s) if visibly clean. Avoid joint areas; wrist and antecubital pit, since they have a higher incidence of mechanical phlebitis. To insert a PVC, 10–15 cm compressor above the puncture site, locate vein by palping, and place the limb in decline to favor venous filling. alcoholic chlorhexidine solution at > 0.5% or aqueous chlorhexidine at 2%. In cases of hypersensitivity, use iodine solutions or alcohol at 70%. PVC is covered with transparent sterile dressing. PVC Maintenance: Reduce connections manipulations; handle stopcocks or bio-connectors with hygienic hand washing and clean gloves.	Category IA **	Recommended ***
Torres-Muñoz et al., 2018 [4]	Vascular Access Nursing Care Recommendations Guide	Clinical Practice Guideline	- Selection of catheter and vein, assess objective, duration, and osmolarity of treatment, state of venous sources, and professional experience. Choose a catheter of the least length and caliber possible, without exceeding vein calibre, to allow blood passage and hemodilution of the preparations. Antiseptic on clean skin, making circles from inside to outside, and let dry. - Evaluate insertion site daily. In case of phlebitis, this is recorded in patient history along with the degree detected. Remove blood-derived administration systems at the end of transfusion. PVC is only changed when clinically justified; there are no benefits on systematic change every 72–96 h.	Category IB **	Recommended ***
Torres-Muñoz et al., 2018 [4]	Vascular Access Nursing Care Recommendations Guide	Clinical Practice Guideline	- It is not necessary to wear sterile gloves as long as aseptic technique is guaranteed. Dressing is placed with clean gloves without touching the adhesive part and without placing tie-shaped adhesive tapes.	Category IC **	Recommended ***
Torres-Muñoz et al., 2018 [4]	Vascular Access Nursing Care Recommendations Guide	Clinical Practice Guideline	- Insert PVC into upper extremities. If bleeding persists after insertion of PVC, absorbent gauze dressing may be placed and fixed until a transparent dressing can be placed. Suture fastening will be avoided. The dressings are changed every 7 days, or if visibly soiled or damp. Dressing change is recorded.	Category II **	Recommended ***
Martínez-Ortega et al., 2019 [7]	Prevention of complications related to peripheral venous catheter for vascular access.	Clinical Practice Guideline	Before inserting a PVC, hand hygiene with alcoholic-based solution or antiseptic soap. Wear clean gloves. Prepare skin with 2% alcoholic chlorhexidine and allow to dry. Cover with sterile, transparent, and semi-permeable dressing to inspect insertion site. Change management systems and connections every 4–7 days, and to reduce risk of infection, clean access port with antiseptic (alcoholic chlorhexidine >0.5% or alcohol at 70%) Remove any PVC that is not essential.	Category IA **	Recommended ***
Martínez-Ortega et al., 2019 [7]	Prevention of complications related to peripheral venous catheter for vascular access.	Clinical Practice Guideline	Select PVC based on objective, expected time of use, and known complications. Select catheter of the smallest caliber and shortest length possible. Avoid areas of the joints (hand, wrist, and antecubital pit), with increased risk of infiltration and injury from extravasation. Dressings will be changed every 7 days, except in pediatric patients, where risk of dislodging PVC is greater than the advantages of changing dressings. Remove PVC if signs of phlebitis, infection, or malfunctioning appear. It is recommended to use The Visual Scale of Phlebitis Assessment (Maddox scale).	Category IB **	Recommended ***
Martínez-Ortega et al., 2019 [7]	Prevention of complications related to peripheral venous catheter for vascular access.	Clinical Practice Guideline	It is recommended to use split septum valves against mechanical ones, which have an increased risk of infection.	Category II **	Recommended ***
Infusion Nurses Society, 2016 [9]	Assessment of the vascular access site, determining type of intervention, education about phlebitis for the patient, and response to treatment	Clinical Practice Guideline	It is recommended to assess our patient’s characteristics regularly, recognize risk factors for bacterial, mechanical, or chemical phlebitis, and consider pharmacological actions, also applying a warm compress and elevating the limb.	Category IB **	Recommended ***

* Joanna Briggs Institute (JBI) degree of evidence (DE) and degree of recommendation (DR), ** Category of HICPAC recommendations, *** The Appraisal of Guidelines for Research and Evaluation (AGREE) Instrument, **** Critical Appraisal Skills Programme Español (CASPe), ***** Berra et al. [24].

**Table 4:** Correspondence with NIC interventions and DE and DR analysis with JBI.

Result	Interventions	Synthesis of the Evidence	NIC [20]
Management and maintenance of intravenous therapy	Protocol monitoring and continuous evaluation	DE*:3c DR*: A	(6520) Health Screening
	Records need to include date of puncture, securement used, professional performing the procedure. When changing the PVC, record date, site, number of puncture attempts, intravenous medication in use.	DE*: 4a DR: A	(4200) Intravenous therapy (i.v.)
	Using sterile saline solution to secure the PVC, to avoid heparin-induced thrombocytopenia.	DE*: 1c DR: A	(2314) Intravascular medication administration (i.v.)
	For intermittent flushing and locking, perform the positive pressure technique to avoid a possible suction effect or backflow when extracting the syringe.	DE*: 2c DR: A	(2314) Intravascular medication administration (i.v.)
	Flushing stopcocks and hubs with normal saline solution. If PVC is used to obtain a blood sample, the use of diluted heparin is indicated.	DR*: B	(4235) Phlebotomy: Cannulated Vessel
	Using double-pump syringes to enable both medication and cleaning solution administration to reduce PVC manipulation and complications.	DE: 1c DR: A	(2314) Intravascular medication administration (i.v.)
	To prevent and treat phlebitis, use Aloe vera, Matricaria chamomilla, or Xianchen.	DE*: 1b DR: A DE*: 1c DR: A	(3584) Skin care: topical treatments
	Removing any PVC that is not essential.	Category IA **	(4200) Intravenous therapy (i.v.)
	Not performing a systematic catheter change every 72 to 96 h. It must be changed when clinically justified.	Category IB **	(4200) Intravenous therapy (i.v.)
	Avoiding insertion into joint areas, wrist, and antecubital fossa.	Category IA **	(4190) Intravenous insertion (i.v.)
	Replacing administration systems, extension cords, and accessories between 4 and 7 days.	Category IA **	(4200) Intravenous therapy (i.v.)
	Guiding patients and family members on signs and symptoms of phlebitis after removing the catheter and at hospital discharge.	DE*: 3c DR*: A DE*: 1ª DR: A	(6610) Risk identification (5510) Health education
Catheter asepsis	Using alcoholic chlorhexidine solution at > 0.5% or aqueous chlorhexidine at 2% to wash skin.	Category IA **	(4200) Intravenous therapy (i.v.)
	Applying antiseptic on clean skin, making circles from inside to outside, and let dry.	Category IB **	(4200) Intravenous therapy (i.v.)
	Handling stopcocks, hubs, ports, and bio-connectors with hygienic hand washing and clean gloves.	Category IA **	(4200) Intravenous therapy (i.v.)
	In cases such as those in emergency areas, replace the catheter as soon as possible if aseptic technique cannot be ensured.	DE: 1c DR: A	(4190) Intravenous insertion (i.v.) (4200) Intravenous therapy (i.v.)
Nursing assessment	Involving the patient in the choice of PVC.	DE*: 3b DR: A	(4190) Intravenous insertion (i.v.) (5510) Health education
	Analysing patient characteristics, prescribed intravenous medications, expected duration of the treatment, and other risk factors for the onset of phlebitis, before opting for a PVC	DE*: 5a DR: A	(6610) Risk identification (2314) Intravascular medication administration (i.v.)
	Assessing osmolarity of treatment and state of venous sources when inserting the PVC.	Category IB **	(4190) Intravenous insertion (i.v.)
	Previously identifying comorbidities such as diabetes mellitus. Educating to provide with knowledge on signs of phlebitis and facilitate early detection and minimize complications.	DE*: 4a DR: A	(6610) Risk identification (5510) Health education
Catheter	Choosing a catheter based on estimated function, duration, and known complications.	DR*: A	(4190) Intravenous insertion (i.v.)
	Choosing a catheter of the least length and caliber possible, without exceeding vein caliber.	Category IB **	(4190) Intravenous insertion (i.v.)
	Using the minimum number of three-way stopcocks. Idle ports should always be capped.	Category IA **	(4200) Intravenous therapy (i.v.)
	Using only one of the ports of the three-way stopcock to place a bio-connector, where intermittent medication solutions and bolus will be administered. Protect with cap infused with alcoholic solution for one use or, if not possible, disinfect the area with alcoholic solution for 30 s.	Category IA **	(4235) Phlebotomy: Cannulated Vessel
Dressing	Using transparent adhesive sterile dressing to achieve frequent and adequate intravenous inspection of the intravenous site.	Category IA **	(4190) Intravenous insertion (i.v.)
	Dressings will be changed every 7 days, except in pediatric patients, where risk of dislodging PVC is greater than the advantages of changing dressings.	Category IB **	(4190) Intravenous insertion (i.v.)
	Using skin glue (cyanoacrylate) at the insertion site to improve catheter securement and reduce rates of phlebitis and occlusion.	DE*: 1c DR: A	(4190) Intravenous insertion (i.v.)
	Using the "I.V. House UltraDressing" in pediatric patients to increase catheter dwell time, and to protect and stabilize PIVCs.	DE*: 1a DR: A	(4190) Intravenous insertion (i.v.)
Nurse as a care prescriber	Applying alternating hot and cold compresses to decrease erythema, edema, and pain.	DE*: 3c DR: A	(1380) Heat/Cold Application
	Applying compresses with 0.9% NaCl to stimulate anti-inflammatory response and relieve pain, redness, swelling, and oedema.	DE*: 2c DR: A	(1380) Heat/Cold Application
	Applying 10 drops (3 mL) of sesame oil (SO) twice daily for two weeks. Massage for 5 min.	DE*: 1c DR: A	(3584) Skin care: topical treatments
	Applying Moist Exposed Burn Ointment (MEBO) for topical treatment of burn injuries.	DE*: 1a DR: A	(3584) Skin care: topical treatments
	Administering chamomile extract (2.5%), as it has anti-inflammatory and anti-edema properties.	DE*: 1b DR: A	(3584) Skin care: topical treatments
	Applying marigold ointment every 8 h for anti-inflammatory effect.	DE*: 1c DR: A	(3584) Skin care: topical treatments
	Applying a compress with Burow solution at a temperature between 2 and 8 degrees Celsius, and leave on for 20 min every 8 h.	DE*: 3c DR: A	(3584) Skin care: topical treatments
Nurse following protocols and guidelines	Applying of anti-inflammatory or corticosteroid agents, and application of a hot and/or wet compress.	DE*: 1a DR: A	(3584) Skin care: topical treatments
	Using corticosteroids and piroxicam to prevent phlebitis.	DE*: 1c DR: A DE*: 1ª DR: A	(2316) Medication Administration: Skin
	Applying topical treatments with aloe vera or "Chamomilla Recutita" using wet compresses at 38 degrees Celsius on the affected area. Apply topical diclofenac and “Essaven” heparin gel for the treatment of phlebitis. Topical products such as notoginseny and 5 mg nitroglycerin patch accelerate the improvement of phlebitis symptoms, as compared to heparinoid substances.	DE*: 1a DR: A	(2316) Medication Administration: Skin (3584) Skin care: topical treatments

* Joanna Briggs Institute (JBI) degree of evidence (DE) and degree of recommendation (DR), ** Category of HICPAC recommendations.

## Data Availability

All data is available within this article.
